# PINK1 phosphorylates ubiquitin predominantly in astrocytes

**DOI:** 10.1038/s41531-019-0101-9

**Published:** 2019-12-11

**Authors:** Sandeep K. Barodia, Laura J. McMeekin, Rose B. Creed, Elijah K. Quinones, Rita M. Cowell, Matthew S. Goldberg

**Affiliations:** 1 Center for Neurodegeneration and Experimental Therapeutics, Department of Neurology, University of Alabama at Birmingham, Birmingham, AL 35294 USA; 2 Department of Neuroscience, Southern Research, Birmingham, AL 35205 USA; 3 Department of Cell, Developmental, and Integrative Biology, University of Alabama at Birmingham, Birmingham, AL 35294 USA; 4 Department of Neurobiology, University of Alabama at Birmingham, Birmingham, AL 35294 USA

**Keywords:** Parkinson's disease, Parkinson's disease, Cellular neuroscience, Experimental models of disease

## Abstract

Loss-of-function mutations in PINK1 are causally linked to recessively inherited Parkinson’s disease (PD), with marked loss of dopaminergic neurons in the substantia nigra that are required for normal movement. PINK1 is a nuclear-encoded mitochondrial-targeted kinase that phosphorylates a conserved serine at amino acid 65 (pS65) in ubiquitin as well as Parkin, another gene with loss-of-function mutations linked to recessive parkinsonism. The steady-state levels of PINK1 protein are very low, even in cells that express PINK1, because PINK1 is normally targeted for degradation after mitochondrial import by a process that is dependent upon mitochondrial membrane potential. Dissipation of the mitochondrial membrane potential with ionophores, such as CCCP and valinomycin, causes the accumulation of PINK1 on the outer mitochondrial membrane, a marked increase of pS65-ubiquitin and the recruitment of Parkin, which targets dysfunctional mitochondria for degradation by autophagy. While the high penetrance of PINK1 mutations establish its critical function for maintaining neurons, the activity of PINK1 in primary neurons has been difficult to detect. Mounting evidence implicates non-neuronal cells, including astrocytes and microglia, in the pathogenesis of both idiopathic and inherited PD. Herein we used both western analysis and immunofluorescence of pS65-ubiquitin to directly compare the activity of PINK1 in primary neurons, astrocytes, microglia, and oligodendrocyte progenitor cells cultured from the brains of wild-type (WT) and PINK1 knockout (KO) rat pups. Our findings that PINK1-dependent ubiquitin phosphorylation is predominantly in astrocytes supports increased priority for research on the function of PINK1 in astrocytes and the contribution of astrocyte dysfunction to PD pathogenesis.

## Introduction

Loss-of-function mutations in the gene encoding *PTEN-induced kinase 1* (PINK1) are causally linked to a recessively inherited form of Parkinson’s disease (PD) clinically similar to idiopathic PD with earlier onset.^1^ The normal function of PINK1 and the mechanisms by which PINK1 mutations cause PD remain areas of active research.^2^ The primary sequence of PINK1 contains a mitochondrial targeting sequence at the N-terminus and a kinase domain homologous to serine/threonine kinases of the calcium/calmodulin family. Known substrates of PINK1 include ubiquitin and the ubiquitin homology domain of Parkin, which are both phosphorylated by PINK1 at a conserved serine at amino acid position 65 (S65).^3–6^ Numerous PD-linked PINK1 mutations have been identified, most of which are point mutations that destabilize the protein or disrupt the kinase activity of PINK.^7,8^ Because PINK1 is apparently the only kinase that phosphorylates ubiquitin, S65-phosphorylated ubiquitin (pS65-ub) can be used as a measure of PINK1 activity.^4,5,9^ Previous in vitro studies have demonstrated that depolarization of mitochondria with ionophores, such as carbonyl cyanide m-chlorophenyl hydrazone (CCCP) or valinomycin, causes the accumulation of PINK1 and the recruitment of Parkin to the outer mitochondrial membrane, which promotes mitochondrial autophagy.^3,10,11^ The translocation of Parkin from the cytosol to the outer mitochondrial membrane is dependent on PINK1 kinase activity.^12–15^ Loss-of-function mutations in Parkin are also causally linked to early onset recessive parkinsonism.^16^ PINK1 activates the E3-ubiquitin ligase activity of Parkin both directly by phosphorylation of Parkin at S65^3,17^ and indirectly by phosphorylation of ubiquitin at S65, which binds to and potently activates Parkin.^4–6^ Postmortem analysis of PINK1-linked PD brains shows loss of dopaminergic neurons in the substantia nigra similar to both Parkin-linked PD and idiopathic PD.^18,19^ Unlike idiopathic PD brains, which by definition have Lewy body pathology in addition to nigral cell loss, some—but not all—autopsy reports of PINK1 and Parkin-linked PD brains show severe nigral cell loss without apparent Lewy body pathology.^20–22^ This suggests that PINK1 and Parkin are required for the long-term survival of dopaminergic neurons irrespective of Lewy body pathology. In vitro studies have shown that PINK1 deficiency decreases the viability of human and mouse dopaminergic neuronal cultures.^23^ In vivo conditional knockdown of PINK1 causes age-dependent loss of dopaminergic neurons in mice.^24^ Together, this genetic, cell biological, biochemical, and neuropathological evidence establishes PINK1 kinase activity as critical for the survival of dopaminergic neurons in the substantia nigra, the loss of which underlies the motor symptoms of PD and characterizes the primary neuropathology of PD, including PINK1 and Parkin-linked PD.^18,21^

PINK1 is widely expressed throughout the brain, including in the substantia nigra,^25,26^ and in many other tissues, with particularly high expression in tissues reliant on mitochondria to meet high energy and metabolic demands, such as skeletal muscle and heart.^27^ Within the brain, PINK1 mRNA is expressed in all the major cell types.^28–30^ A major unanswered question is the extent to which PINK1 protein functions in neurons compared to non-neuronal cells, such as astrocytes, microglia, and oligodendrocytes. Recent data implicating dysfunction of astrocytes and microglia in PD initiation and progression underscores the increasing importance of answering this question.^31–33^ Therefore, we systematically examined the activity of PINK1 in cultured primary neurons, astrocytes, microglia, and oligodendrocyte progenitor cells (OPCs) using both western analysis and immunofluorescence with antibodies specific for ubiquitin phosphorylated at S65 (pS65-Ub). Parallel cultures were derived from PINK1 knockout (KO) rats to confirm the specificity of the pS65-Ub signal as a measure of PINK1 activity.

## Results

### Derivation and analysis of primary neurons and glia

**Fig. 1:**
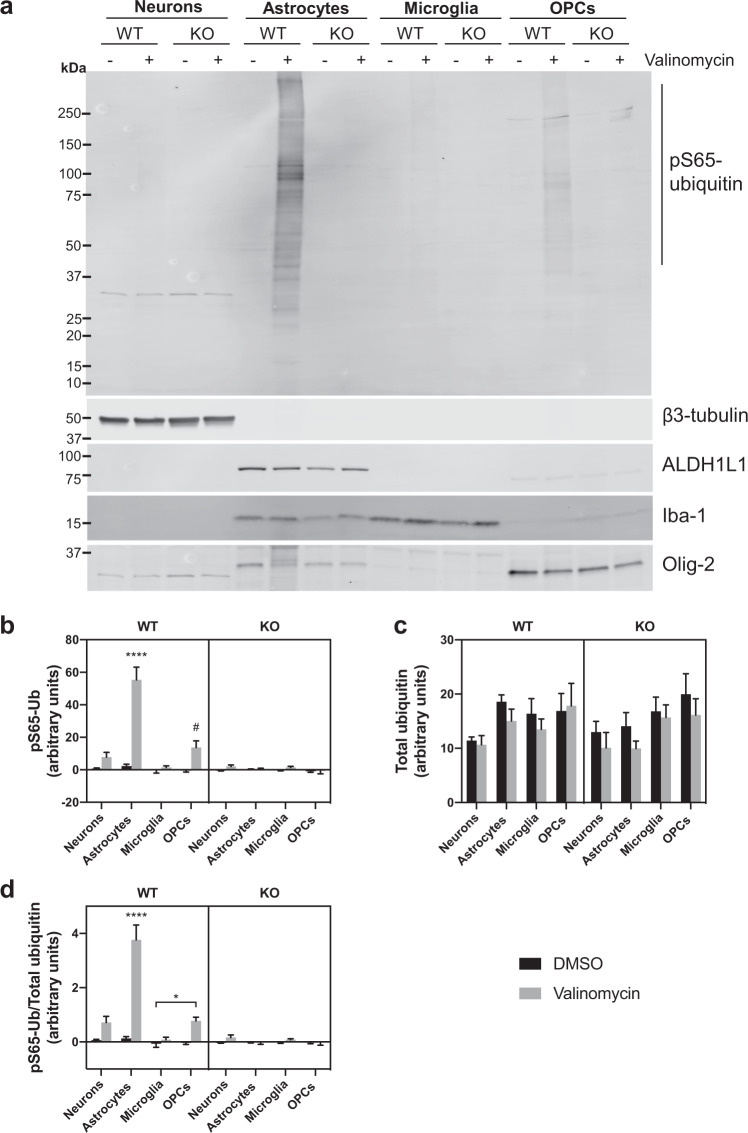
Western analysis of cell lysates. **a** Western analysis of primary neurons, astrocytes, microglia, and OPCs derived from WT and PINK1 KO rats. Cultured cells were treated with 100 nM valinomycin or DMSO control for 4 h at 37 °C, then harvested for western analysis. Ten micrograms of cell lysate protein was loaded in each lane, resolved by SDS-PAGE, transferred to PVDF membrane, and blotted using anti-pS65-Ub antibody. The same membrane was re-probed using the antibodies indicated on the lower panels as markers for neurons (β3-tubulin), astrocytes (ALDH1L1), microglia (Iba-1), and OPCs (Olig-2). **b** Mean +/− SEM pS65-Ub level measured by densitometry of western blot membranes from four independent experiments. (Four asterisks (****) indicate significantly different from all other groups by ANOVA followed by Tukey’s multiple comparisons test *p* < 0.001; hash (#) indicates significantly different from all other groups except valinomycin-treated WT and KO neurons and microglia, as well as DMSO-treated WT astrocytes *p* < 0.05). **c** Mean +/− SEM total ubiquitin levels from the same four membranes. **d** Mean +/− SEM pS65-Ub level normalized to the total ubiquitin level in each lane (Four asterisks (****) indicate significantly different from all other groups *p* < 0.0001; asterisk (*) indicates *p* < 0.05).

Primary cultures were derived from wild-type (WT) and PINK1 KO rat pups using protocols and culture conditions optimized to yield separate cultures enriched for neurons, astrocytes, microglia, and OPCs (see “Methods”). For each culture, cells from the same tube were plated both on six-well plates for western analysis and on glass coverslips for confocal analysis. Neurons were allowed to mature for 14 days prior to treatment. Astrocytes, microglia, and OPCs were allowed to proliferate for 7–10 days until they reached >70% confluency prior to treatment. For western analysis, cells in six-well plates were treated in parallel for 4 h with either 100 nM valinomycin dissolved in dimethyl sulfoxide (DMSO) or DMSO alone as a control. Western analysis of cell lysates using an antibody specific for pS65-Ub showed a high molecular weight smear typical of poly-ubiquitinated proteins only in cells from WT rats treated with valinomycin to induce PINK1 activity (Fig. 1a). To confirm the cell types and protein loading, the same membrane was re-probed with antibodies specific for the neuronal marker β3-tubulin, the astrocytes marker ALDH1L1, the microglial marker Iba-1, and the OPC marker Olig-2 (Fig. 1a lower panels). Only background pS65-Ub signal was observed in the lanes from cells treated with DMSO control and in all the lanes from cells derived from PINK1 KO rats, confirming that PINK1 is the only kinase that apparently phosphorylates ubiquitin at serine 65 in the presence of valinomycin and confirming the specificity of the pS65-Ub antibody used for western analysis (Fig. 1a). Remarkably, valinomycin treatment induced PINK1 activity predominantly in astrocytes. This was consistently observed in western blots from three additional independent experiments (Supplementary Fig. 1). Densitometry of the pS65-Ub level in each lane of western blots from four independent experiments followed by three-way analysis of variance (ANOVA) with genotype, treatment, and cell type as factors showed a significant main effect for each factor and significant interactions among all the factors (*p* < 0.0001 for all). Tukey’s multiple comparisons test showed that only valinomycin-treated WT astrocytes were significantly different from all other groups (*p* < 0.0001, asterisks in Fig. 1b). In addition, the pS65-Ub level in the valinomycin-treated WT OPCs was significantly different from all but five of the other groups: valinomycin-treated WT and KO neurons and microglia, as well as DMSO-treated WT astrocytes (*p* < 0.05, hash in Fig. 1b).

To determine the extent to which differences in total ubiquitin levels contributed to the differences in pS65-Ub levels, we performed densitometry on the same four membranes probed with an antibody specific for total ubiquitin, utilizing a separate channel from the pS65-Ub antibody in the Licor scanner (Fig. 1c and Supplementary Fig. 1). Three-way ANOVA of the total ubiquitin levels showed a significant main effect of cell type (*p* < 0.001) but no significant main effect of genotype or treatment and no significant interactions. Tukey’s multiple comparisons test showed no significant differences in total ubiquitin levels in any of the 120 possible pairwise comparisons.

Upon normalizing the pS65-Ub level to the total ubiquitin level in each lane (Fig. 1d), three-way ANOVA showed a significant main effect for each factor and significant interactions among all the factors (*p* < 0.0001 for all). Similar to the pS65-Ub level prior to normalization to total ubiquitin, Tukey’s multiple comparisons tests showed that only valinomycin-treated WT astrocytes were significantly different from all other groups (*p* < 0.0001, asterisks in Fig. 1d). Only one other pairwise comparison was statistically significant: the normalized pS65-Ub level in the valinomycin-treated WT OPCs was significantly different from DMSO-treated WT microglia (*p* < 0.05, asterisk in Fig. 1d). Some of the pS65-Ub signal in valinomycin-treated OPCs could be attributed to contaminating astrocytes because the astrocyte marker, ALDH1L1, appears faintly in the OPC lanes (Fig. 1a). The neuronal marker β3-tubulin appears exclusively in the neuronal cultures; however, the microglial marker Iba-1 also appears in the astrocyte lanes (Fig. 1a).

**Fig. 2:**
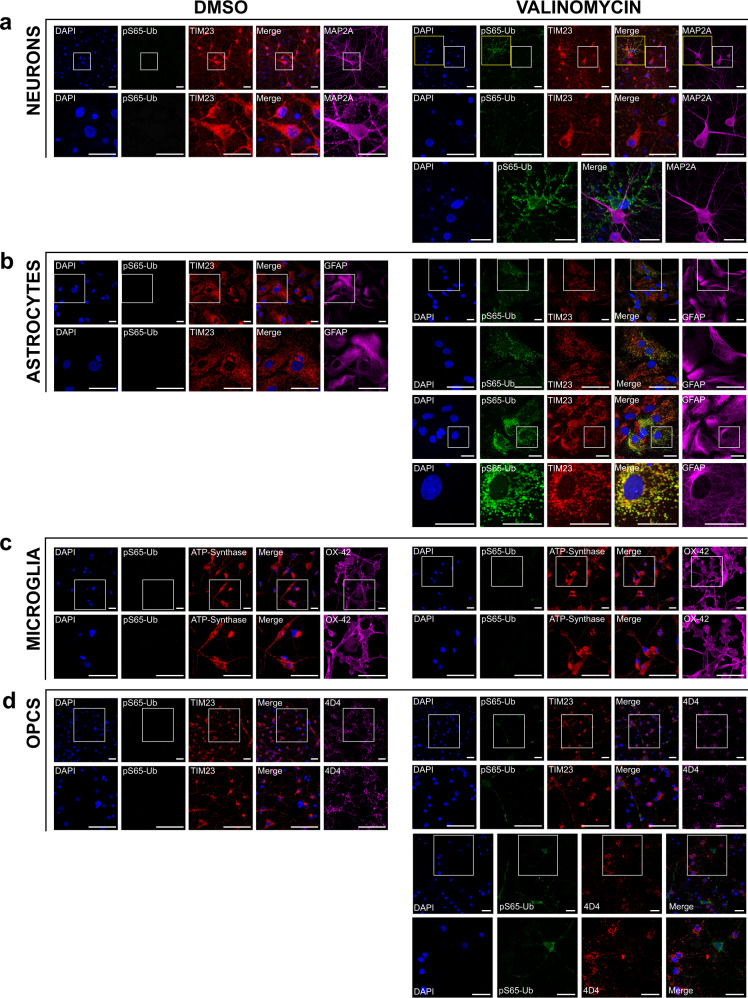
Immunofluorescence of primary neurons, astrocytes, microglia, and OPCs derived from WT rats. Cultured cells were treated with 100 nM valinomycin or DMSO control for 4 h at 37 °C, then fixed and analyzed by immunofluorescence with the indicated antibodies. Cells were also stained with DAPI to show cell nuclei. White boxes indicate areas shown at higher magnification immediately below. Each channel is shown individually as well as an image of the DAPI, pS65-Ub, and mitochondrial marker channels merged, as indicated. **a** Primary cortical neurons treated with DMSO control (left) or with valinomycin (right). Higher-magnification images of the areas indicated by yellow boxes are shown at bottom to clarify the non-overlapping pS65-Ub immunofluorescence with the neuronal marker MAP2A. **b** Primary cortical astrocytes treated with DMSO control or with valinomycin. A second panel of higher-magnification images of cortical astrocytes treated with valinomycin is included to allow better visualization of the colocalization of pS65-Ub with the mitochondrial marker TIM23 in astrocytes. **c** Primary cortical microglia treated with DMSO control or with valinomycin. The mitochondrial marker ATP-Synthase was substituted for TIM23 because the microglial marker OX-42 antibody is the same species and isotype as anti-TIM23. **d** Primary cortical OPCs treated with DMSO control or with valinomycin. Additional higher-magnification images of cortical OPCs treated with valinomycin are shown below to better visualize the absence of colocalization of pS65-Ub and the OPC marker 4D4. Data are representative of results from four independent experiments. All scale bars are 25 microns.

To further assess the relative enrichment of each culture and to localize the pS65-Ub immunoreactivity at the cellular and subcellular level, we analyzed the cells plated on coverslips from each culture using confocal microscopy and immunofluorescence with antibodies specific for pS65-Ub as a measure of PINK1 activity, TIM23 as a marker for mitochondria, microtubule‐associated protein 2A (MAP2A) as a marker for neurons, glial fibrillary acidic protein (GFAP) as a marker for astrocytes, OX-42 as a marker for microglia, and 4D4 as a marker for OPCs (Fig. 2). Consistent with the western blot data, pS65-Ub immunoreactivity was only observed in cells treated with valinomycin to induce PINK1 activity and not in cells treated with DMSO control (Fig. 2). The specificity of the pS65-Ub antibody for immunofluorescence was confirmed by the absence of immunofluorescence in cells derived from PINK1 KO littermates cultured and immunolabeled in parallel with the cells derived from WT pups (Supplementary Fig. 2). The relative enrichment for each culture was quantified by counting the number of marker-labeled neurons, astrocytes, microglia, and OPCs in 16 fields captured by a ×20 objective epifluorescence microscope near the center of each coverslip from 3 independent cultures of each cell type. As shown in Supplementary Fig. 3, the cultures of neurons, microglia, and OPCs were highly enriched with only a small ~1% contamination of astrocytes. However, the astrocyte cultures averaged ~76% astrocytes, ~19% microglia, and ~6% OPCs.

In primary neuronal cultures, no pS65-Ub immunofluorescence was observed in DMSO-treated cells while valinomycin treatment induced clear pS65-Ub immunofluorescence in sparse cells (Fig. 2a). However, none of the pS65-Ub immunofluorescence colocalized with the neuronal marker MAP2A, indicating that PINK1 activity was induced only in non-neuronal cells (Fig. 2a lower right panel).

In primary astrocyte cultures, no pS65-Ub immunofluorescence was observed in DMSO-treated cells (Fig. 2b). By contrast, valinomycin treatment of astrocyte cultures induced prominent pS65-Ub immunofluorescence in GFAP-positive cells (Fig. 2b). Moreover, the pS65-Ub immunofluorescence in valinomycin-treated astrocytes co-localized with the mitochondrial marker TIM23 (Fig. 2b lower right panel). pS65-Ub immunofluorescence was only observed in GFAP-immunolabeled cells indicating that PINK1 activity is predominantly in astrocytes.

In primary microglia cultures, no pS65-Ub immunofluorescence was found in DMSO control-treated cells and almost no pS65-Ub immunofluorescence was found in valinomycin-treated cells (Fig. 2c). Essentially all the 4,6-diamidino-2-phenylindole (DAPI)-positive cells colocalized with the microglial marker OX-42, indicating that the cultures were highly enriched for microglia.

In primary OPC cultures, no pS65-Ub immunofluorescence was observed in DMSO-treated cells while valinomycin treatment induced modest pS65-Ub immunofluorescence in sparse cells that did not colocalize with the OPC marker 4D4 (Fig. 2d).

We used the NIS Elements software to quantify the pS65-Ub immunofluorescence intensity per cell in epifluorescence images of coverslips from three independent cultures of each cell type. One-way ANOVA followed by Dunnett’s multiple comparisons test showed a significant effect of cell type (*p* < 0.0001) and significant differences between each cell type except for neurons compared to OPCs (Supplementary Fig. 4). The mean pS65-Ub immunofluorescence of astrocytes was significantly greater than neurons, microglia, and OPCs (*p* < 0.0001). Also, microglia had significantly less pS65-Ub immunofluorescence compared to neurons and OPCs (*p* < 0.05). As shown in Fig. 2, some pS65-Ub immunofluorescence likely originated from sparse astrocytes that contaminated the neuronal and OPC cultures but not the microglial cultures.

To assess the extent to which our findings could be attributed to differences in PINK1 transcript abundance, we used quantitative PCR (qPCR) to measure PINK1 transcript levels in each cell type. RNA isolated from cultures of WT neurons, astrocytes, microglia, and OPCs, together with KO astrocytes as a specificity control, was reverse transcribed and the cDNA was used as template for Taqman qPCR with commercial Taqman primers specific for rat PINK1 and for β-actin control for normalization. PINK1 signal was significantly reduced in KO astrocytes, demonstrating specificity of the PINK1 primer set (Supplementary Fig. 5a). Comparison of PINK1 transcript abundance across cell types revealed significant differences among all cell types (one-way ANOVA, post hoc Tukey’s; *p* < 0.01), with the highest expression in neurons and astrocytes. Therefore, the astrocyte-specific pS65-Ub immunoreactivity we detected by both western analysis and immunofluorescence does not appear to be due to enrichment of PINK1 transcript in astrocytes with respect to rat primary neurons and OPCs.

## Discussion

The most prominent neuropathological feature of PD is the striking loss of dopaminergic neurons in the substantia nigra, although neurodegeneration occurs in many other brain regions and cell types.^34^ Consequently, PD research has been focused on understanding the root cause of this neurodegeneration and on developing neuroprotective therapies, which remain unfulfilled goals. Research on the role of astrocytes and other non-neuronal cells in PD pathogenesis has been relatively eclipsed by research specifically on neurons. Moreover, the functions of PINK1 and Parkin have been most commonly investigated in cell lines, such as HeLa and HEK293, in which mitochondrial autophagy can be more readily observed. The importance of this study is the revelation that valinomycin-stimulated PINK1 activity is induced predominantly in astrocytes compared to neurons, microglia, and OPCs, which suggests that astrocytes may be a key experimental system in which to decipher why PINK1 deficiency invariably leads to PD-related neurodegeneration.

Several previous studies suggest that PINK1 has important functions in astrocytes. Primary astrocytes derived from PINK1 KO mice have increased pro-inflammatory cytokines and higher nitric oxide production upon stimulation of the innate immune response with lipopolysaccharide plus interferon-γ.^35^ The same study found reduced expression of the anti-inflammatory cytokine interleukin-10 from primary microglia derived from PINK1 KO mice compared to WT. This suggests that PINK1 deficiency alters inflammatory gene expression in both astrocytes and microglia, either directly or indirectly via cytokine signaling from other cells. Other studies have shown that PINK1 deficiency impairs both the formation of GFAP-positive astrocytes during development and the proliferation of astrocytes upon stimulation with epidermal growth factor or fetal bovine serum (FBS).^36,37^ However, humans with PINK1-linked PD show elevated basal ganglia myoinositol levels by magnetic resonance spectroscopy, indicative of astrocyte proliferation within the basal ganglia.^38^

For practical reasons, including obtaining enough cells for western analysis, our study was limited to cortical cells. Although we found that PINK1 activity in neurons was below that which could be detected with our methods, it remains possible that PINK1 is active in different neurons or in other brain regions, such as midbrain dopaminergic neurons. Furthermore, our study was limited to activation of PINK1 by depolarizing mitochondria with valinomycin and it remains possible that PINK1 can be activated in neurons using alternative methods. We obtained similar results depolarizing mitochondria with 10 μM CCCP instead of valinomycin and using treatment times up to 16 h (Supplementary Fig. 6), but we selected 0.1 μM valinomycin and a treatment time of 4 h to minimize potential confounding effects of toxicity, which we observed at higher doses and longer treatment times. Notably, pS65-Ub has been reported in human fibroblasts directly converted into induced neurons and in primary mouse neurons following treatment with 1 μM valinomycin for 12 h and using custom generated anti-pS65-Ub antibodies.^9^ PINK1 activity in neurons is also supported by phos-tag gel detection of phosphorylated Parkin in mouse primary neurons treated with 30 μM CCCP for 3 h.^39^ Although it was not statistically significantly above the controls by densitometry (Fig. 1b–d), our western analysis showed faint pS65-Ub signal in valinomycin-treated WT neurons (Fig. 1 and Supplementary Fig. 1). This is consistent with our quantification of pS65-Ub by immunofluorescence using a different anti-pS65-Ub antibody (Supplementary Fig. 4). We used the best anti-pS65-Ub antibodies we could procure, which only worked for western analysis or for immunofluorescence, not both. Therefore, our western analysis and immunofluorescence represent two independent analyses of the same primary cell cultures without using the same antibody.

Our results do not exclude the possibility that PINK1 is active in other non-neuronal cells, such as microglia, especially under different conditions or activation states.^31^ Moreover, we derived cultures of each cell type at only a single stage in development, which was selected as the optimum for cell viability, yield, and proximity to the developmental stage of the other cell types, as well as the ability to derive all four cell types from the same brain. It has been shown that PINK1 expression increases substantially during embryonic development and upon differentiation of neural stem cells into neurons.^23,36^ In addition, PINK1 expression in the mouse cortex, hippocampus, and striatum increases markedly with aging.^40^ Therefore, further studies are warranted to determine the extent to which the activity of PINK1 varies with developmental stage, cellular differentiation, and aging.

Our qPCR analysis of rat PINK1 mRNA levels was limited to a single developmental stage and was intended to determine the extent to which the differences we observed in PINK1 activity could be due to differences in PINK1 expression. Our data showing higher abundance of PINK1 mRNA in neurons than in astrocytes suggests that the activity of PINK1 does not correspond, necessarily, with transcript abundance. Human and rodent brain cell transcriptomic databases generally show the expression of PINK1 in all these cell types but to different extents depending on brain region, age, or treatment.^28,29,41^ Together, this suggests that the activity of PINK1 could be regulated posttranscriptionally in a cell-type-specific manner or astrocytes could respond differently to valinomycin treatment compared to neurons, microglia, and OPCs.

Despite the inherent differences between in vitro and in vivo systems as well as potential species differences between the expression and functions of rat and human PINK1, our findings suggest that PINK1 has one or more vital functions in astrocytes that are disrupted by human PINK1 mutations causally linked to PD. Whether it is the loss of PINK1 function specifically in astrocytes that causes PINK1-linked PD and whether this function is altered in astrocytes in idiopathic PD remain important questions to be answered. Immunohistochemical analysis of postmortem human brains shows PINK1 protein in all cell types and all brain regions.^42^ The regional, cellular, and subcellular pattern of PINK1 immunoreactivity appears indistinguishable between neuropathologically normal and sporadic PD brains.^42^ Human PINK1 immunoreactivity has a granular appearance in both neuronal and non-neuronal cell types; however, reactive astrocytes show strong diffuse PINK1 staining.^42^ Prominent PINK1 immunoreactivity colocalizes with reactive astrocytes near amyloid plaques and vascular deposits in Alzheimer’s disease brains as well as reactive astrocytes in active demyelinating lesions characteristic of multiple sclerosis (MS) brains; however, anti-PINK1 only weakly stains astrocytes in chronic inactive MS lesions.^43^ This has prompted the suggestion that PINK1 is upregulated in reactive astrocytes as a protective mechanism when astrocytes are stressed.^43^ Gene expression profiling indicates that cultured primary rat astrocytes, such as those in this study, are more comparable to reactive astrocytes relative to their resting state in vivo because the process of dissociating and culturing cells in vitro results in an “activated” pattern of gene expression.^44^ Therefore, our results from primary rat cells may reflect PINK1 activity in reactive astrocytes more than resting-state astrocytes in vivo. Nevertheless, we could detect PINK1 activity only after ionophore treatment, indicating that the process of deriving and culturing cells is not sufficient to significantly induce PINK1 activity, as measured by anti-pS65-Ub.

Overall, our results necessitate greater investigation of the function of PINK1 in astrocytes to ascertain the cellular and molecular mechanisms by which PINK1 deficiency leads to PD. Furthermore, our findings add to the growing evidence implicating astrocyte dysfunction in PD pathogenesis.^32,33^ Our data suggest that a better understanding of the function of PINK1 in astrocytes is needed to facilitate the development of neuroprotective therapies for PINK1-linked PD and potentially idiopathic PD.

## Methods

### Chemicals

Valinomycin (Sigma V0627) was dissolved in DMSO at a stock concentration of 10 mM, then diluted in cell culture media to a final concentration of 100 nM resulting in a final DMSO concentration of 0.001%.

### Primary antibodies

#### For western analysis

Anti-phospho-ubiquitin (ser65) (E2J6T) rabbit monoclonal antibody (mAb; Cell Signaling #62802, 1:1000), mouse anti-total ubiquitin (Santa Cruz #sc-8017, 1:200), β3-Tubulin (D71G9) rabbit mAb (Cell Signaling #5568S, 1:5000), rabbit anti-Iba1 [EPR16588] (Abcam #ab178846, 1:2000), rabbit anti-olig2 (Novus Biologicals #NBP128667SS, 1:1000), and mouse anti-ALDH1L1 N103/39 (Antibodies incorporated #75-140, 1:1000).

#### For immunocytochemistry

Mouse monoclonal anti-MAP2A (EMD Millipore #MAB378, 1:500), anti-phospho-ubiquitin (ser65) (E5T1W) rabbit mAb (Cell Signaling #70973, 1:500), mouse anti-TIM23 (BD Transduction Laboratories #611223, 1:500), anti-ATP synthase beta mouse mAb 3D5AB1 (Invitrogen #A-21351, 1:500), anti-GFAP (GA5) mouse mAb (Cell Signaling #3670, 1:500), mouse anti-CD11b/c, clone OX-42 (BD Biosciences #554859, 1:500), and mouse mAb 4D4 (Developmental Studies Hybridoma Bank #mAb 4D4(A2B5-like)-s, 1:500).

### Cell culture

Cortical primary neurons and glia were derived as follows from postnatal day 0 (P0) and day 3 (P3) pups, respectively, from PINK1 KO and WT Long Evans rats (Horizon Discovery #TGRL4690). This line has been shown to have a 26 base pair targeted deletion in rat PINK1 exon 4, causing a reading frame shift and premature stop codon.^45^ All use of animals was reviewed and approved by the UAB Institutional Animal Care and Use Committee.

### Primary cortical neuron culture

Rat pups (P0) were isolated from dams and quickly decapitated. Heads were dunked in 70% ethanol for 5 min to prevent microbial contamination from cage bedding, then transferred to a fresh tube containing ice-cold Hank’s Balanced Salt Solution (HBSS, Life Technologies #14175-095). Using sterilized fine scissors and forceps, cortices were dissected in ice-cold HBSS on a petri dish. After meninges were removed, cortices were transferred to a fresh 15-ml tube of ice-cold HBSS. The buffer was aspirated and cortices were digested in papain (Worthington Biochemical #LK003178) at 37 °C for 22 min. Papain was neutralized by washing the tissue in warm neurobasal media (Neurobasal-A media, B27 supplement, 10% FBS, 5 mM L-Glutamine, and 100 units penicillin/streptomycin solution, all from Life Technologies). Cortices were triturated using fire-polished glass pipettes, 3 times starting with large-bore size, then medium-bore size, then small-bore size to dissociate the digested cortical tissue. The supernatant containing cells was filtered through a 70-µm cell strainer in a sterile 50-ml tube and then centrifuged at 1000 × *g* for 5 min at room temperature. The supernatant was aspirated carefully and the cell pellet was re-suspended in neurobasal media. The cell density was determined using a hemocytometer, then 2 × 10^6^ cells/well were seeded in 6-well plates. In parallel, 3 × 10^5^ cells/well were seeded in 24-well plates containing poly-D-lysine-coated glass coverslips. Cells were maintained in a 5% CO_2_ 37 °C humidified incubator. After 4 h, the media was completely replaced with fresh neurobasal media without FBS. After 3 days in vitro (DIV3), half the media was changed with fresh 37 °C neurobasal media containing 1 μM cytosine arabinoside to inhibit the proliferation of non-neuronal cells. Half the media was subsequently changed every 2–3 days. After DIV14, neurons were treated with 100 nM valinomycin or DMSO control for 4 h.

### Astrocyte culture

Astrocyte-enriched cultures were derived by immunoisolation (Miltenyi magnetic beads conjugated to anti-GLAST antibodies, Cat#130-095-825) following the manufacturer’s instructions and published protocols.^46^ Briefly, cortices were harvested from P3 rat pups as above and incubated in freshly prepared papain solution at 37 °C for 22 min. Tissues were triturated using fire-polished Pasteur pipettes and filtered through a 40-μm cell strainer. The flow through was centrifuged at 1000 × *g* for 5 min at room temperature and the pellet was re-suspended in ice-cold column Buffer (1× phosphate-buffered saline (PBS) pH 7.4, 0.5% bovine serum albumin, and 2 mM EDTA) and centrifuged at 1000 × *g* for 5 min at 4 °C. The pellet was re-suspended in ice-cold column buffer (25 μl per 1 × 10^6^ cells) in a sterile 1-ml tube. To the cell suspension, 2 μl per 1 × 10^6^ cells of anti-GLAST (ACSA-1)-Biotin was added and gently inverted to mix. Cells were incubated at 4 °C for 10 min on a rotating mixer at slow speed. Cells were washed with 1 ml of column buffer followed by centrifugation at 1000 × *g* for 5 min at 4 °C. The pellet was re-suspended in fresh column buffer (25 μl per 1 × 10^6^ cells) and 2 μl anti-Biotin MicroBeads were added per 1 × 10^6^ cells and gently inverted to mix. The cell suspension was incubated at 4 °C for 20 min on a rotating mixer at slow speed, then centrifuged at 1000 × *g* for 5 min at 4 °C. Cells were washed with 1 ml column buffer followed by centrifugation at 1000 × *g* for 5 min at 4 °C. The pellet was re-suspended in astrocyte culture medium (Dulbecco's Modified Eagle Medium (DMEM) GlutaMAX, 10% FBS, 2mM L-Glutamine, 100 units penicillin/streptomycin solution, all from Life Technologies) and cells were counted with a hemocytometer. In all, 2 × 10^6^ cells/well were plated on 6-well plates and 1 × 10^5^ cells/well were plated on 24-well plates containing glass coverslips. Cells were maintained in a 5% CO_2_ 37 °C humidified incubator, changing the media every 2–3 days. When astrocytes were >70% confluent (7–10 days), cells were treated with 100 nM valinomycin or DMSO control for 4 h.

### Microglia culture

To obtain pure microglial cultures, astrocytes were derived as above but without immune-isolation and plated in 75-cm culture flasks with loose non-ventilated caps and allowed to proliferate until ~70% confluent (5–7 days), then complete astrocyte media was replaced with microglia media (DMEM/F12, 10% FBS, 2 mM L-Glutamine, 100 units penicillin/streptomycin, all from Life Technologies, supplemented with 50 ng/ml granulocyte macrophages colony-stimulating factor from Peprotech) to allow proliferation of microglia, as described in the published protocols.^47^ Once microglia became >70% confluent, flasks were tightly closed and sealed in a sterile plastic bag, then rotated in a 37 °C incubator at 150 rpm for 45 min to shake off microglia. Media containing detached microglia was filtered through a 40-μm cell strainer in a 50-ml tube and the filtrate was centrifuged at 1000 × *g* for 5 min at room temperature. The pellet was re-suspended in microglia media and the cell concentration was counted with a hemocytometer. In all, 2 × 10^6^ cells/well were plated on 6-well plates and 1 × 10^5^ cells/well were plated on 24-well plates containing glass coverslips. When the microglia were >70% confluent (~2–3 days after re-plating), microglia were treated with 100 nM valinomycin or DMSO control for 4 h.

### OPC culture

OPC-enriched cultures were derived using immunoisolation (Miltenyi magnetic beads conjugated to anti-A2B5 antibodies, Cat#130-095-825) following the manufacturer’s instructions and adapting the published protocols.^48,49^ Briefly, cortices were harvested from P3 rat pups as described for astrocytes, except anti-A2B5 was used instead of anti-GLAST. The cell pellet was re-suspended in modified OPC culture media (DMEM/F12, 2% B27 supplement, 1% N2 supplement, 1% Insulin Transferrin Selenium solution, 10 ng/ml platelet-derived growth factor-AA, 10 ng/ml basic fibroblast growth factor, 100 units penicillin/streptomycin solution, all from Life Technologies) and cells were counted using a hemocytometer. In all, 2 × 10^6^ cells/well were plated on 6-well plates and 1 × 10^5^ cells/well were plated on 24-well plates with coverslips. The media was completely changed every other day. After the cells were >70% confluent (~7 days), OPCs were treated with 100 nM valinomycin or DMSO control for 4 h.

### Quantification of culture enrichment for each cell type

To estimate the relative abundance of each cell type in each culture, coverslips of fixed cells were immunolabeled as described below with markers of each cell type (MAP2A for neurons, GFAP for astrocytes, OX-42 for microglia, and 4D4 for OPCs) as well as DAPI for nuclei. From each of the 3 different cultures of each cell type, images of 16 fields near the center of each coverslip were collected using a ×20 objective on a Nikon Ti-S epifluorescence microscope with a motorized stage. In each field, the total number of neurons, astrocytes, microglia, and OPCs were counted manually and tabulated. A mean of 799 cells were counted for each coverslip (range of 442–1441). For each of the three different cultures of each of the four different cell types, the relative percentage of each cell type was calculated from the tabulated cell counts, then the mean and SEM of the three cultures of each cell type were calculated.

### Western analysis

Treated cells in 6-well plates were harvested by aspirating the media and scraping the cells in 1 ml PBS, followed by centrifugation at 1000 × *g* for 5 min at room temperature. Cell pellets were re-suspended in Triton lysis buffer (50 mM Tris pH 7.4, 150 mM NaCl and 1% Triton X-100) containing cocktails of protease inhibitors (Sigma P8340) and phosphatase inhibitors (Sigma P5726, P0044). Cells were incubated on ice for 30 min followed by trituration for 7 times using a 25-gauge needle. Lysed cells were centrifuged at 17,000 × *g* for 10 min at 4 °C. Supernatants were transferred to fresh Eppendorf tubes and protein concentration was measured using a BCA Assay Kit (Pierce). Ten micrograms of protein (diluted in 2× Laemmli buffer with 5% β-mercaptoethanol) was resolved using 4–20% sodium dodecyl sulfate-polyacrylamide gel electrophoresis at 150 volts for 1 h. Proteins were transferred to a polyvinylidene difluoride membrane in Tris-Glycine buffer overnight (14–18 h) at a constant current of 100 mAmp. The membrane was incubated in blocking buffer (Licor Odyssey) for 1 h at room temperature. Primary antibodies were diluted in 25% Licor Odyssey buffer in 1× PBST (1× PBS with 0.01% Tween 20). Membranes were incubated in primary antibody solutions overnight at 4 °C with gentle shaking. Membranes were washed 3 times for 5 min each wash in 1× PBST. Corresponding secondary antibodies (IgG-IR680/IR800) were added at a dilution of 1:10,000 in 1× PBST and incubated for 2 h at room temperature. Membranes were washed 3 times for 5 min with 1× PBST and scanned using a Licor clx scanner for simultaneous infra-red color detection of two proteins of interest. Membranes were subsequently re-probed by western analysis, as above, with antibodies specific for each cell-type marker protein.

### Quantification of western blots

Protein levels were quantified using NIH ImageJ densitometry tools (integrated intensity) with background subtraction. For each lane, the region from 100 to 250 kilodaltons (kDa) was analyzed to avoid the mouse anti-ALDH1L1 marker protein band that appears just below 100 kDa only in the astrocyte lanes of the mouse anti-ubiquitin blots. Densitometry was performed on western blots from four independent experiments, and the levels of total ubiquitin, pS65-Ub, and pS65-Ub/total ubiquitin were analyzed by three-way ANOVA with genotype, cell type, and treatment as factors. Tukey’s multiple comparisons test was used for pairwise comparisons.

### Immunocytochemistry

After treatment, cells grown on glass coverslips were rinsed once with 1× PBS and fixed in 10% Formalin (buffered saline, pH 7.4 Fisher SF100) for 20 min at room temperature and permeabilized in 0.1% triton X-100 in 1× PBS for 15 min at room temperature with gentle shaking followed by blocking in 10% normal donkey serum and 10% normal goat serum in 1× PBS for 1 h. Cells were incubated with primary antibodies (diluted in 0.02% triton X-100, 1% normal donkey serum, and 1% normal goat serum in 1× PBS) overnight at 4 °C. Cells were washed 3 times for 5 min each in 1× PBS. Corresponding secondary antibodies conjugated to Alexa Fluor 488/546/647 (diluted in 0.02% triton X-100, 1% normal donkey serum, and 1% normal goat serum in 1× PBS) were added to the cells and incubated for 2 h at room temperature with gentle shaking. Cells were washed 3 times for 5 min each in 1× PBS and coverslips were mounted on glass microscope slides using Prolong Diamond antifade media with DAPI (Life Technologies P36971). To minimize systematic error, immunocytochemistry was conducted on WT and KO cells +/− valinomycin at the same time with the same antibody cocktail and fluorescence images were acquired during the same session with the same image acquisition settings.

### Confocal microscopy

High-resolution high-power images of cells were acquired with a Leica TCS SP5 laser scanning confocal microscope using a ×63 oil-immersion objective. Serial *Z*-stacks of 6–10 images were acquired at 0.7-μm intervals (scan averaged four times; 1024 × 1024 pixels). Maximum intensity projection images were generated from *Z*-stack planes using the NIH ImageJ software. For each experiment, treated and control WT and KO cultures of each cell type were imaged during the same session with identical acquisition parameters including laser power, pinhole, digital gain, and noise reduction.

### Quantification of pS65-Ub immunofluorescence

After valinomycin treatment, coverslips from three separate cultures of each cell type were processed as above and imaged with identical acquisition settings using a ×20 objective on a Nikon Ti-S epifluorescence microscope for pS65-Ub immunofluorescence together with markers for each cell type and DAPI for nuclei. Random fields were captured near the center of each coverslip using a motorized stage. Nikon NIS Elements software was used to measure the mean pS65-Ub immunofluorescence intensity per cell for the following numbers of cells from 3 independent experiments for each culture: neurons: 46, 42, 52; astrocytes: 27, 29, 33; microglia: 39, 47, 44; OPCs: 39, 43, 54. Data were analyzed by one-way ANOVA with cell type as the independent variable followed by Dunnett’s multiple comparisons test.

### Quantitative PCR analysis of PINK1 mRNA levels

Total RNA was isolated from cultures of WT neurons (*n* = 4), WT astrocytes (*n* = 9), KO astrocytes (*n* = 12), WT OPCs (*n* = 4), and WT microglia (*n* = 8) using the TRIzol/chloroform–isopropanol method according to the manufacturer’s instructions (Thermo Fisher). RNA concentration and purity were assessed using a NanoDrop One (Thermo Fisher). Equivalent amounts of RNA (1 μg) were treated with DNase I (Promega) at 37 °C for 30 min followed by DNase Stop solution at 65 °C for 15 min. RNA was then reverse transcribed using the High-Capacity cDNA Archive Kit (Applied Biosystems). Samples were then diluted 1:3 and transcript levels were quantified using the rat-specific primers for Pink1 (Rn01518847_m1) or beta-actin (Rn00667869_m1) from Applied Biosystems and JumpStart Taq Readymix (Sigma). The PCR protocol used had an initial ramp (2 min, 50 °C; 10 min, 95 °C) and 40 subsequent cycles (15 s, 95 °C; 1 min, 60 °C). Relative transcript concentration was calculated using the calibrator method, in comparison to a standard curve generated from pooled samples from WT astrocyte cultures and then diluted (1:5, 1:10, 1:20, 1:40). Pink1 transcript was normalized to beta-actin, analyzed by one-way ANOVA followed by Tukey’s multiple comparisons, and expressed as ratio to control samples +/−SEM.

### Statistical analysis

Data were analyzed and plotted using GraphPad Prism version 8.

### Reporting summary

Further information on experimental design is available in the Nature Research Reporting Summary linked to this article.

## Supplementary information

Supplementary Figures

Reporting Summary

## Data Availability

The data for this study are available from the corresponding author on reasonable request.
