# Supplementary material for: PINK1 phosphorylates ubiquitin predominantly in astrocytes
Source: NPJ Parkinsons Dis. 2019 Dec 11;5:29. doi: 10.1038/s41531-019-0101-9 (PMC6906478; doi:10.1038/s41531-019-0101-9)
Supplement: Supplementary file 1 — Supplementary Figures [file 41531_2019_101_MOESM1_ESM.pdf]

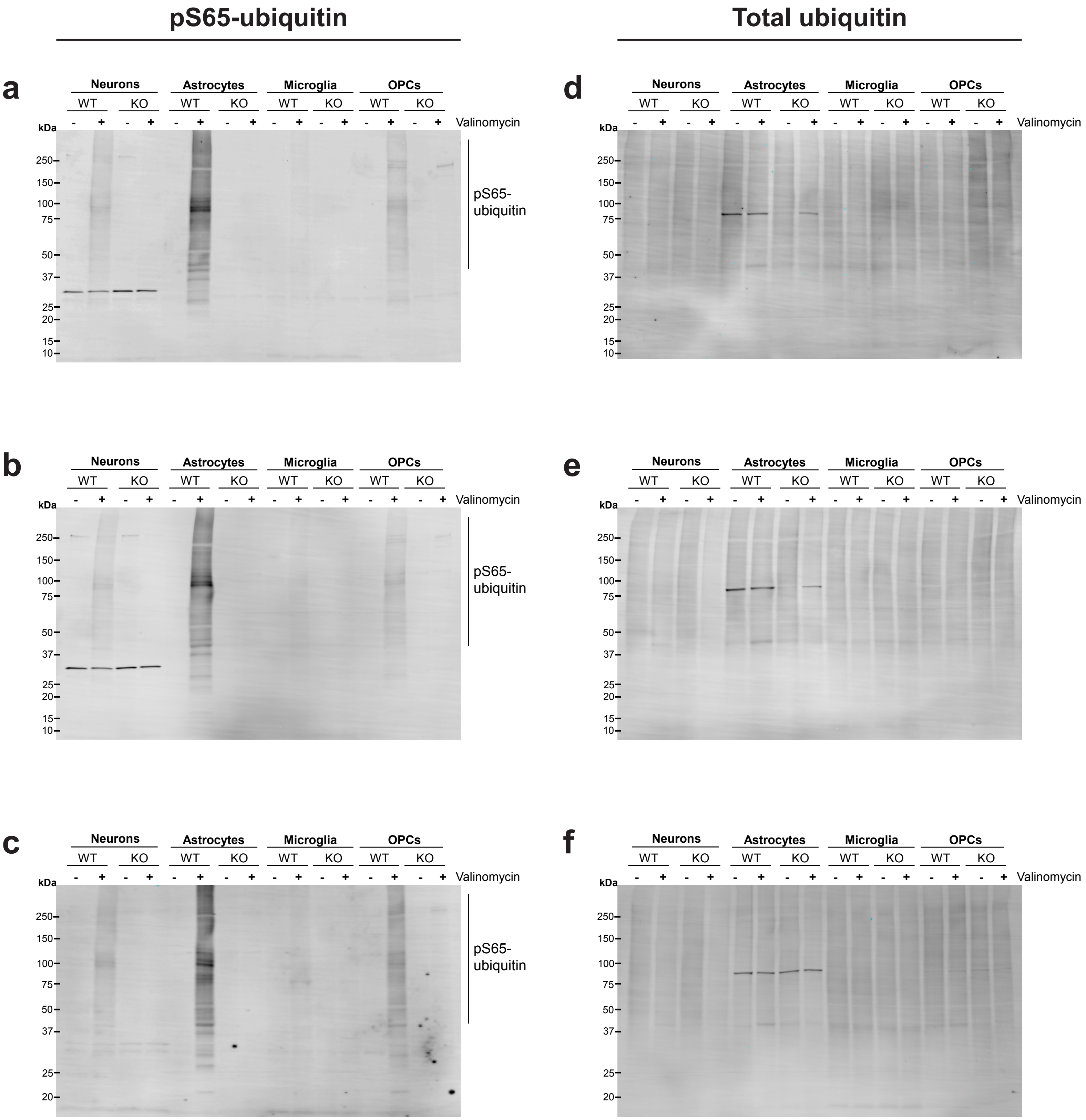

Supplementary Figure 1 Western blots from 3 additional independent experiments conducted as shown in Figure 1. **a-c** Anti-pS65-ubiquitin western analysis of valinomycin or DMSO control treated primary neurons, astrocytes, microglia and OPCs derived from WT and PINK1 KO rats. **d-f** Anti-total ubiquitin western analysis of the same membranes shown in a-c. The band appearing only in the astrocyte lanes just below 100 kDa is from the mouse anti-ALDH1L1 antibody that was used as an astrocyte-specific marker prior to re-probing the membrane with mouse anti-total ubiquitin.

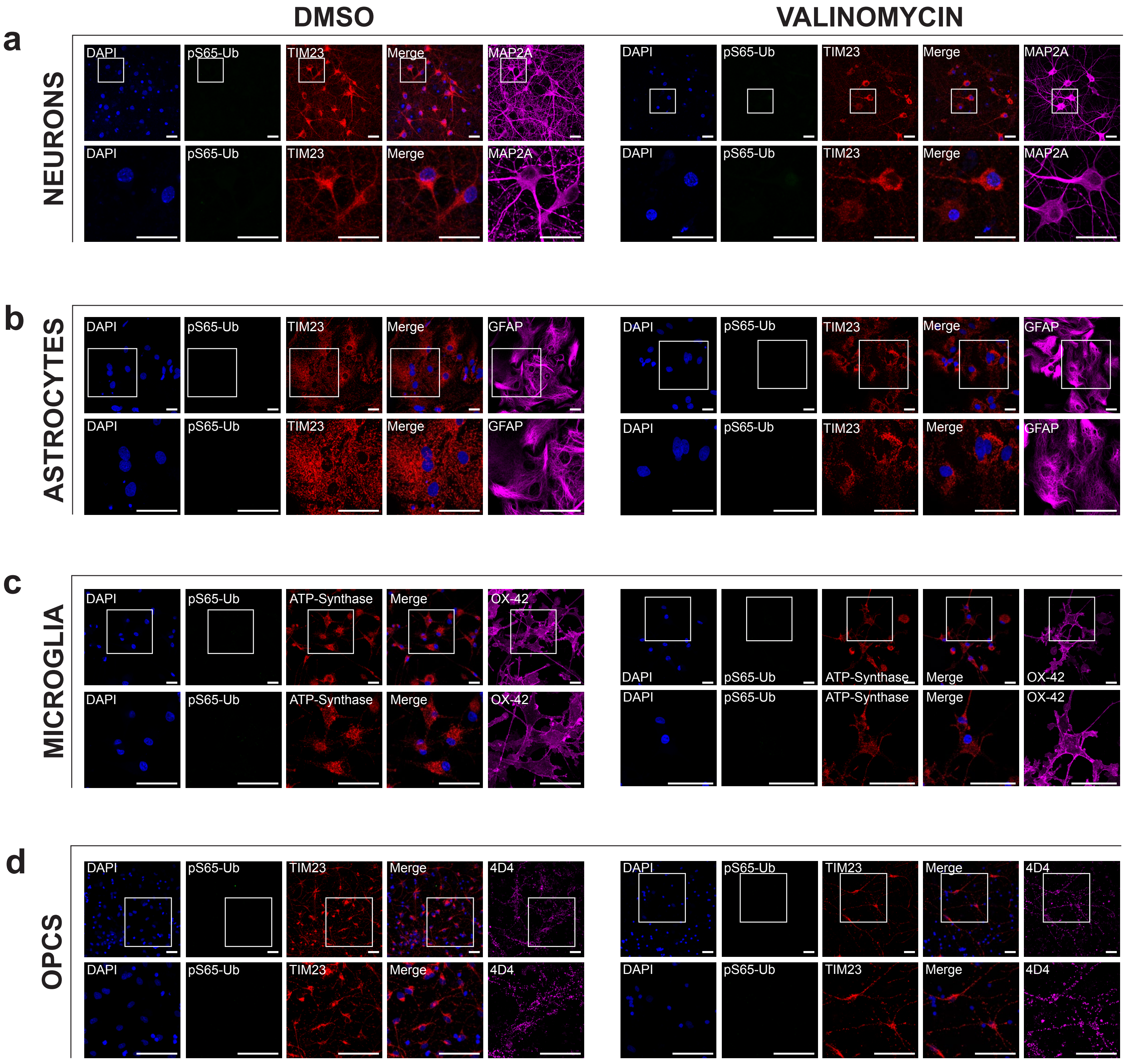

Supplementary Figure 2 Immunofluorescence of primary neurons, astrocytes, microglia and OPCs derived from PINK1 KO rats. Cultured cells were treated with 100 nM valinomycin or DMSO control for 4 hours at 37°C, then fixed and analyzed by immunofluorescence with the indicated antibodies. Cells were also stained with DAPI to show cell nuclei. Boxes indicate areas shown at higher magnification below. **a** Primary cortical neurons treated with DMSO control or valinomycin. **b** Primary cortical astrocytes treated with DMSO control or valinomycin. **c** Primary cortical microglia treated with DMSO control or valinomycin. **d** Primary cortical OPCs treated with DMSO control or valinomycin. All scale bars are 25 microns.

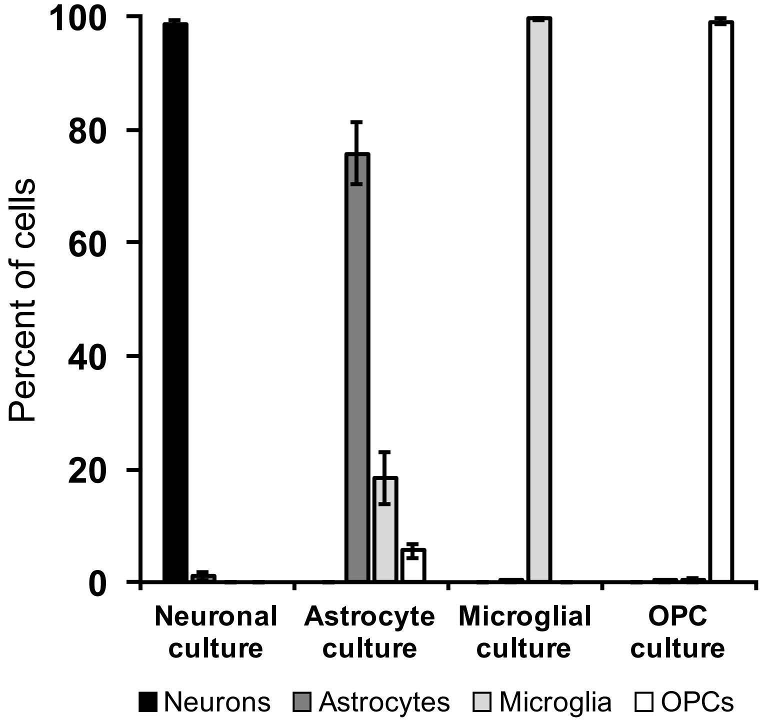

Supplementary Figure 3 Relative abundance of each cell type in each culture. Bars represent mean +/- SEM percent of each cell type in each culture counted in images from 3 independent cultures of each cell type. For each culture, coverslips were immunolabeled with markers of each cell type and 16 fields near the center of each coverslip were imaged using a 20x objective on an epifluorescence microscope with a motorized stage. In each field, the total number of neurons, astrocytes, microglia and OPCs were counted manually and tabulated. A mean of 799 cells were counted for each coverslip (range of 442-1441). Neuronal cultures were 98.9 +/-1.1% neurons and 1.1 +/-0.7% astrocytes. Astrocytes cultures were 75.9 +/-5.5% astrocytes, 18.6 +/-4.6% microglia and 5.6 +/-1.2% OPCs. Microglial cultures were 99.7 +/-0.2% microglia and 0.3 +/- 0.2% astrocytes. OPC cultures were 99.2 +/-0.4% OPCs, 0.4+/-0.2% astrocytes and 0.4 +/-0.3% microglia.

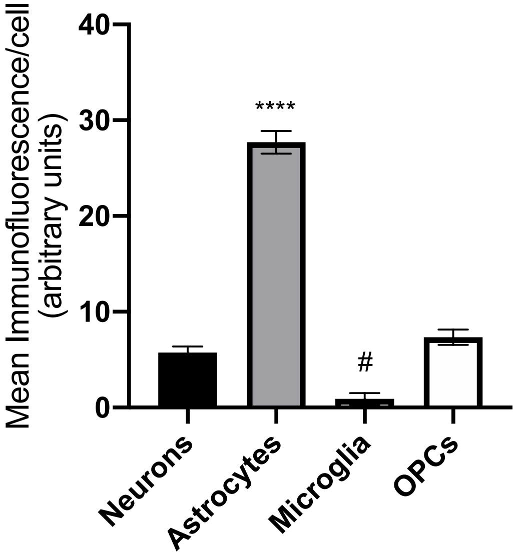

Supplementary Figure 4 Quantification of pS65-Ub immunofluorescence in each cell type. Bars represent mean +/- SEM pS65-Ub immunofluorescence intensity per cell for the following numbers of cells from 3 independent experiments for each culture: neurons: 46, 42, 52; astrocytes: 27, 29, 33; microglia: 39, 47, 44; OPCs: 39, 43, 54. One-way ANOVA followed by Dunnett's multiple comparisons test showed a significant effect of cell type ($p<0.0001$) and significant differences between each cell type except for neurons compared to OPCs. The mean pS65-Ub immunofluorescence of astrocytes was significantly greater compared to neurons, microglia and OPCs (**** $p<0.0001$) while microglia had significantly less pS65-Ub immunofluorescence compared to neurons and OPCs (# $p<0.05$).

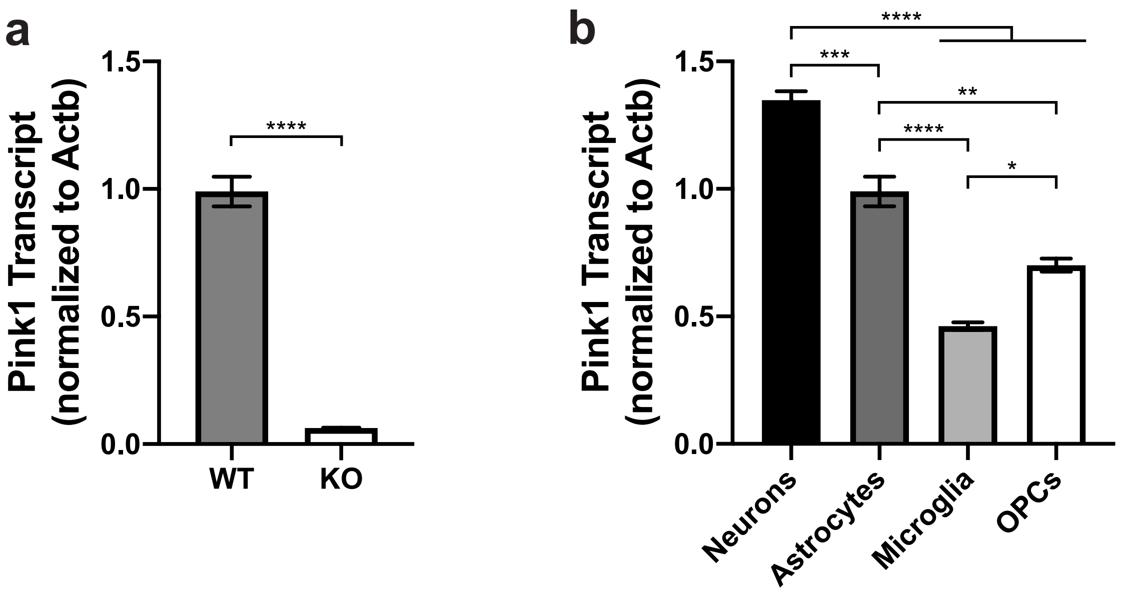

Supplementary Figure 5 Quantitative PCR measurement of PINK1 transcript levels in cDNA prepared from cultures of WT neurons (n=4), WT astrocytes (n=9), WT microglia (n=8), WT OPCs (n=4), and KO astrocytes (n=12), using primers specific for rat PINK1 normalized to β-actin (Actb). **a** PINK1 transcript abundance is significantly reduced in PINK1 KO compared to WT astrocytes, confirming the specificity of the Taqman PCR primer set (**** $p<0.0001$, two-tailed t-test). **b** Relative levels of rat PINK transcript in WT neurons, astrocytes, microglia and OPCs normalized to β-actin. (**** $p<0.0001$; *** $p<0.001$; ** $p<0.01$; * $p<0.05$ ANOVA followed by Tukey's multiple comparisons tests). Bars represent mean +/- SEM expression level compared to WT astrocytes.

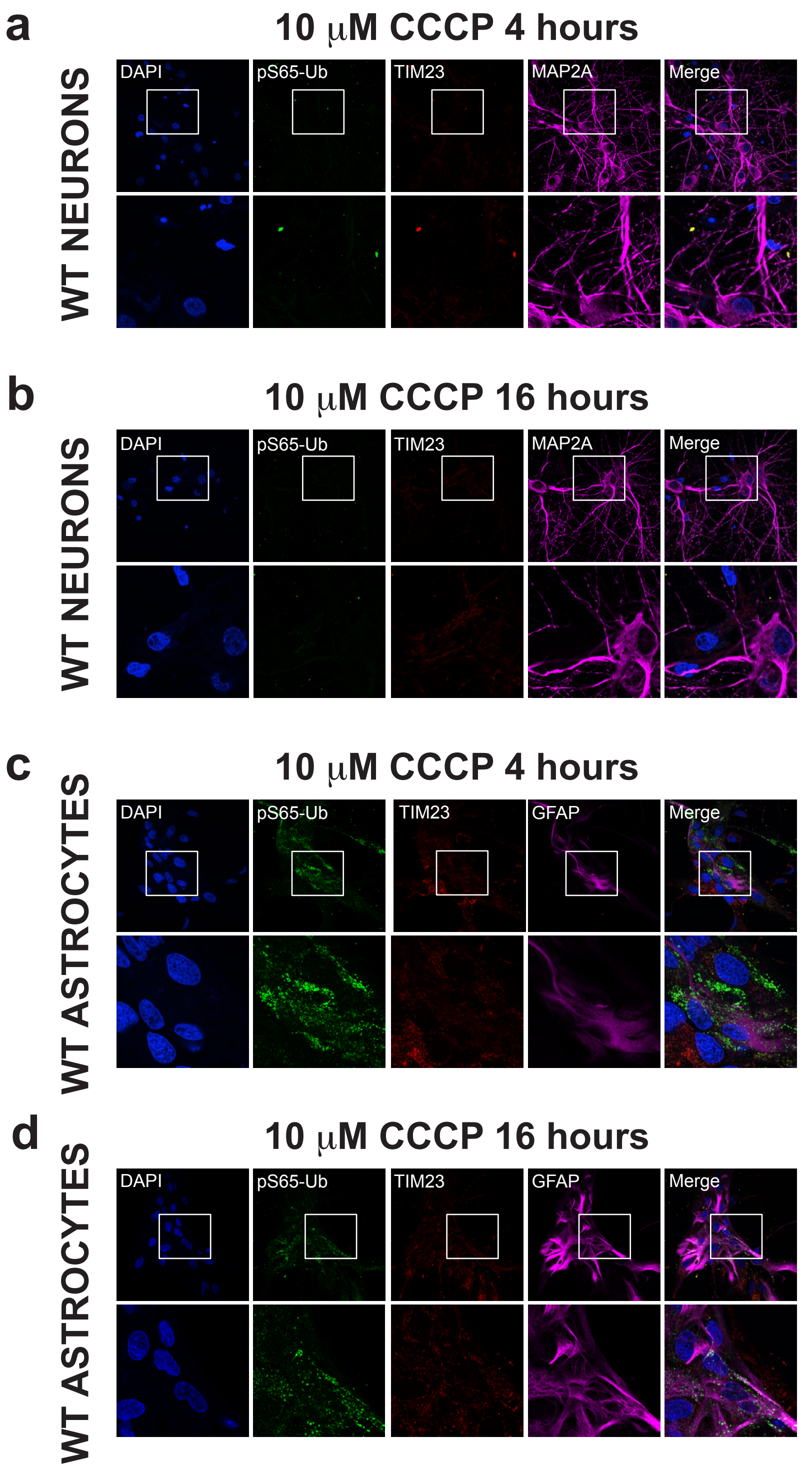

Supplementary Figure 6 Representative images of WT neurons **(a-b)** and astrocytes (c-d) treated with 10 μM CCCP for 4 hours **(a, c)** or 16 hours (b, d), then fixed and analyzed by immunofluorescence with the indicated antibodies. Cells were also stained with DAPI to show cell nuclei. Boxes indicate areas shown at higher magnification below.
